# Design and validation of novel flow cytometry panels to analyze a comprehensive range of peripheral immune cells in mice

**DOI:** 10.3389/fimmu.2024.1432816

**Published:** 2024-08-13

**Authors:** Ainara Barco-Tejada, Rocio López-Esteban, Francisca Mulero, Marjorie Pion, Rafael Correa-Rocha, Manuel Desco, Lorena Cussó

**Affiliations:** ^1^ Departamento de Bioingeniería, Universidad Carlos III de Madrid, Madrid, Spain; ^2^ Unidad de Medicina y Cirugía Experimental, Instituto de Investigación Sanitaria Gregorio Marañón, Madrid, Spain; ^3^ Laboratorio de Inmuno-regulación, Unidad de Medicina y Cirugía Experimental, Instituto de Investigación Sanitaria Gregorio Marañón, Madrid, Spain; ^4^ Unidad de Imagen Molecular, Centro Nacional de Investigaciones Oncológicas (CNIO), Madrid, Spain; ^5^ Unidad de Imagen Avanzada, Centro Nacional de Investigaciones Cardiovasculares Carlos III (CNIC), Madrid, Spain; ^6^ CIBER de Salud Mental, Instituto de Salud Carlos III, Madrid, Spain

**Keywords:** mice, longitudinal studies, immune system, flow cytometry-methods, peripheral blood

## Abstract

The use of flow cytometry in mice is constrained by several factors, including the limited availability of mouse-specific antibodies and the need to work with small volumes of peripheral blood. This is particularly challenging for longitudinal studies, as serial blood samples should not exceed 10% of the total blood volume in mice. To address this, we have developed two novel flow cytometry panels designed to extensively analyze immune cell populations in mice during longitudinal studies, using only 50 µL of peripheral blood per panel. Additionally, a third panel has been designed to conduct a more detailed analysis of cytotoxic and inhibitory markers at the end point. These panels have been validated on a lipopolysaccharide (LPS)-induced lung inflammation model. Two experiments were conducted to 1) validate the panels’ sensitivity to immune challenges (*n*=12) and 2) to assess intrinsic variability of measurements (*n*=5). In both experiments, we collected 50 µL of peripheral blood for each cytometry panel from the maxillary venous sinus. All antibodies were titrated to identify the optimal concentration that maximized the signal from the positive population while minimizing the signal from the negative population. Samples were processed within 1 hour of collection using a MACSQuant Analyzer 16 cytometer. Our results demonstrate that these immunological panels are sensitive enough to detect changes in peripheral blood after LPS induction. Moreover, our findings help determine the sample size needed based on the immune population variability. In conclusion, the panels we have designed enable a comprehensive analysis of the murine immune system with a low blood volume requirement, enabling the measure of both absolute values and relative percentages effectively. This approach provides a robust platform for longitudinal studies in mice and can be used to uncover significant insights into immune responses.

## 1 Introduction

Flow cytometry is a fluorescence-based technique for analyzing the optical properties of cells and quantifying various cellular markers when they are suspended in a fluid stream. This method allows for the simultaneous analysis of multiple cellular markers, making it invaluable for both clinical (1) and research applications (2). Flow cytometry plays a crucial role in diagnosing and monitoring the progression of numerous immunological disorders (3–5), and is also widely used in preclinical research (2).

In preclinical studies, mice are the most commonly used animal model. Despite sharing many immunological markers with humans (6, 7), the use of flow cytometry in mice presents several challenges that complicate cytometry panel design. These challenges include: (a) limited availability of mouse-specific antibodies and fluorochromes compared to the broader range available for non-spectral cytometers (8); (b) the need to work with small volumes of peripheral blood, which complicates longitudinal studies due to the limitation on blood draws not exceeding 10% of the mouse’s total blood volume (9–11); and (c) variability introduced by methodological factors such as anesthesia and animal handling (12, 13).

Due to these limitations, most flow cytometry studies in rodents focus on isolated organs, especially the spleen, because of its ease of extraction and high concentration of immune cells (10, 14). This has resulted in fewer studies investigating the peripheral blood immune landscape comprehensively, with most studies focusing on simple panels or specific cell populations (15–17). Furthermore, stress induced by animal handling can significantly affect immune outcomes and increase intersubject variability, complicating longitudinal studies (12). To mitigate these effects, it is recommended to use anesthesia during blood extraction and to introduce a handling period before experimental procedures (12).

In this paper, we propose two novel cytometry panels designed for comprehensive analysis of immune cell populations in mice in longitudinal studies, using only 100 μL of blood (50 μL for each panel). Additionally, a third panel was designed to perform detailed analysis of cytotoxic and inhibitory markers at the study endpoint. These panels have been validated in a lipopolysaccharide (LPS) lung inflammation model.

## 2 Materials and methods

### 2.1 Peripheral blood flow cytometry panels design

We selected fourteen specific antibodies, each one for a specific immune marker (**Tables 1**, **2**) in 3 different cytometry panels: Myeloid panel, Lymphoid panel and Intracellular panel.

**Table 1:** Antibodies used in the myeloid and lymphoid panels.

Myeloid Panel					
Name	Channel	Comercial brand	Volume in 50µL	Reference	Clone
B220	APC5	Biolegend	0.5µL	103231	RA3-6B2
CCR2	PC7	Miltenyi	1µL	130-120-818	REA538
CD11b	PC5.5	Miltenyi	0.5µL	130-113-809	REA592
CD11c	APC7	Miltenyi	1µL	130-110-704	REA754
CD172a	VioBlue	Miltenyi	0.5µL	130-123-151	REA1201
CD45	VioGreen	Miltenyi	1µL	130-123-900	30F11
CD49b	ECD/PE-Vio615	Miltenyi	0.5µL	130-116-437	REA981
Ly6C	PerCP/PC5	Biolegend	0.7µL	128028	HK1.4
CD80	APC	Miltenyi	1µL	130-116-461	REA983
CD86	APC	Miltenyi	2.5µL	130-102-558	PO3.3
F4/80	FITC	Miltenyi	0.5µL	130-117-509	REA126
Ly6G	BV605	Biolegend	0.7µL	127639	1A8
MHC-II	BV650	Biolegend	0.7µL	107641	M5/114.15.2
Siglec-F	PE	Miltenyi	1µL	130-112-174	REA798
Lymphoid Panel					
Name	Channel	Comercial brand	Volume in 50µL	Reference	Clone
CCR4	PE	Biolegend	2.5µL	131204	2G12
CCR6	BV605	Biolegend	2.5µL	129819	29-2L17
CD138	PC7	Miltenyi	1µL	130-102-318	REA104
CD19	BV570	Biolegend	1µL	115535	6D5
CD25	FITC	Miltenyi	1µL	130-120-088	REA568
CD3	VioBlue	Miltenyi	1µL	130-118-849.	17A2
CD4	BV650	Biolegend	1.25µL	100545	RM4-5
CD44	APC5	Biolegend	0.5µL	103026	IM7
CD45	VioGreen	Miltenyi	1µL	130-123-900.	30F11
CD62L	PerCP/PC5	Biolegend	0.7µL	104410	MEL-14
CD8	ECD/PE-Vio615	Miltenyi	1µL	130-123-914	REA601
CXCR3	APC7	Biolegend	0.7µL	126540	CXCR3-173
NK1.1	APC	Miltenyi	1µL	130-120-507	REA1162
TNF-RII	PC5.5	Miltenyi	2µL	130-104-701	REA228

The table shows the fluorophore and clone for each antibody, as well as the commercial brand and reference. None of the antibodies in these panels is mouse strain-restricted.

**Table 2:** Antibodies used in the intracellular panel.

Name	Channel	Comercial brand	Volume in 300 µL (10^6^ cells)	Reference	Clone
Viability	VioBlue	ebiosciencie	1 µL	65-0863-18	
CD3	VioGreen/BV510	Biolegend	2.5 µL	100233	17A2
NK1.1	BV570	Biolegend	5 µL	108733	PK136
CD25	BV605	Biolegend	5 µL	102035	PC61
CD4	BV650	Biolegend	2.5 µL	100545	RM4-5
Granzyme B	FITC	Biolegend	5 µL	372205	QA16A02
FOXP3	PE	Miltenyi	5 µL	130-111-678	REA788
CD8	ECD/PE-Vio615	Miltenyi	1 µL	130-123-914	REA802
TIM-3	PerCP/PC5	RD Systems	7.5 µL	FAB1529C	215008
PD1	PC5.5	Miltenyi	1 µL	130-111-957	REA802
CTLA4	PC7	Biolegend	2.5 µL	106313	UC10-4B9
Perforina	APC	Biolegend	5 µL	154303	S16009A
CD44	APC5	Biolegend	1 µL	103026	IM7
CCR5	APC7	Miltenyi	1 µL	130-120-168	REA354

The table shows the fluorophore and clone for each antibody, as well as the commercial brand and reference. Except for NK1.1 (which is not valid for AKR, BALB/c, CBA/J, C3H, DBA/1, DBA/2, NOD, SJL, and 129 strains) the rest of the antibodies are not mouse strain-restricted.

This selection for each panel was done considering the clone, the 14 available fluorochromes with its spectral overlap and the antigenic density of the immune marker (**Tables 1**, **2**).

All antibodies were titrated to determine their concentration (**Supplementary Tables 1**, **2**), achieving an optimal balance between signal level for positive populations and negative populations.

### 2.2 Sample collection

Two cytometry panels were used to assess immune populations in peripheral blood: The Myeloid Panel and the Lymphoid Panel. Peripheral blood samples were collected from the maxillary venous sinus using a sterile lancet. A total of 100 µL of peripheral blood was collected per animal, with 50 µL used for each panel, and stored in 500 µL ethylenediaminetetraacetic acid (EDTA) tubes (VACUTEST). Before blood collection, mice were anesthetized with 3% sevoflurane in 100% oxygen to minimize stress and discomfort. After each blood draw, mice received an intraperitoneal injection of 100 µL of saline to replace the extracted volume.

In the validation experiment all mice were sacrificed at the endpoint through exsanguination via intracardiac puncture (using 4% sevoflurane in 100% oxygen). At this point, 300 µL of blood was collected in EDTA tubes for an additional cytometry panel—the Intracellular Panel —designed to analyze transcription factors and cytotoxic proteins such as granzyme B and perforin.

### 2.3 Sample processing

All samples were processed within one hour of extraction to ensure data accuracy. Data acquisition was performed using a MACSQuant Analyzer 16 cytometer (Miltenyi Biotec, Bergisch Gladbach, Germany).

**Figure 1:**
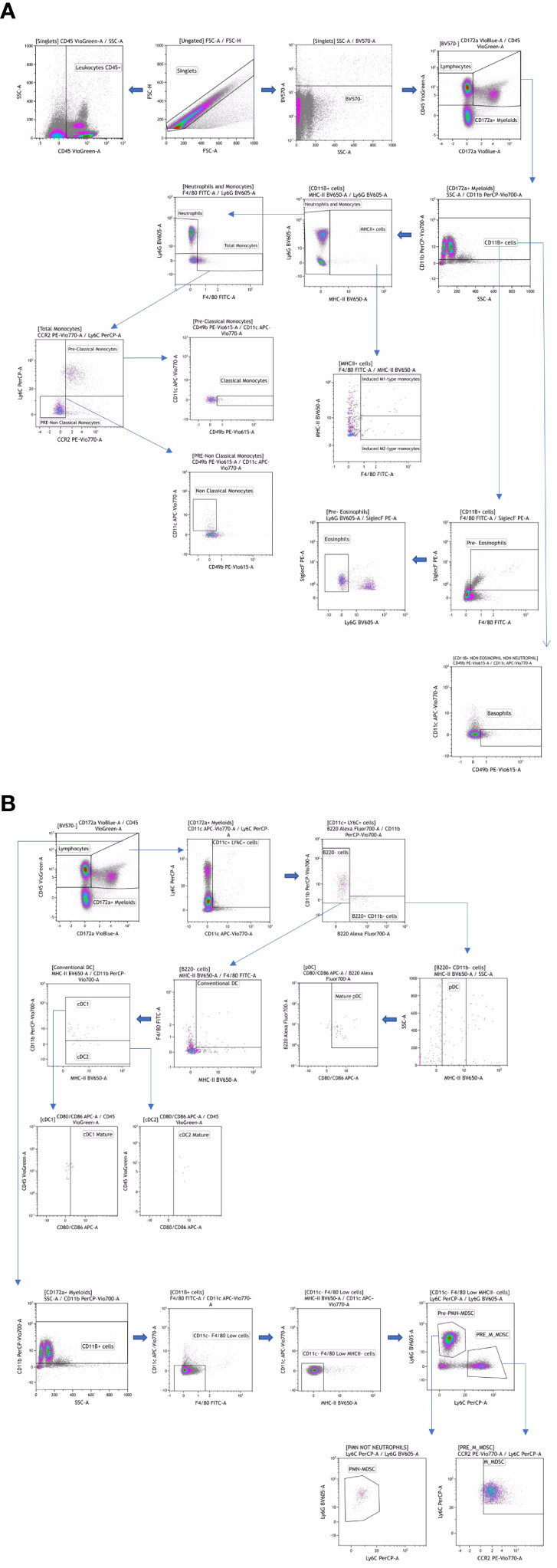
Manual gating strategy by differential expression of extracellular markers for the myeloid panel **(A, B)**. Representative examples of flow cytometry plots determined on whole blood labeled from one individual.

**Figure 2:**
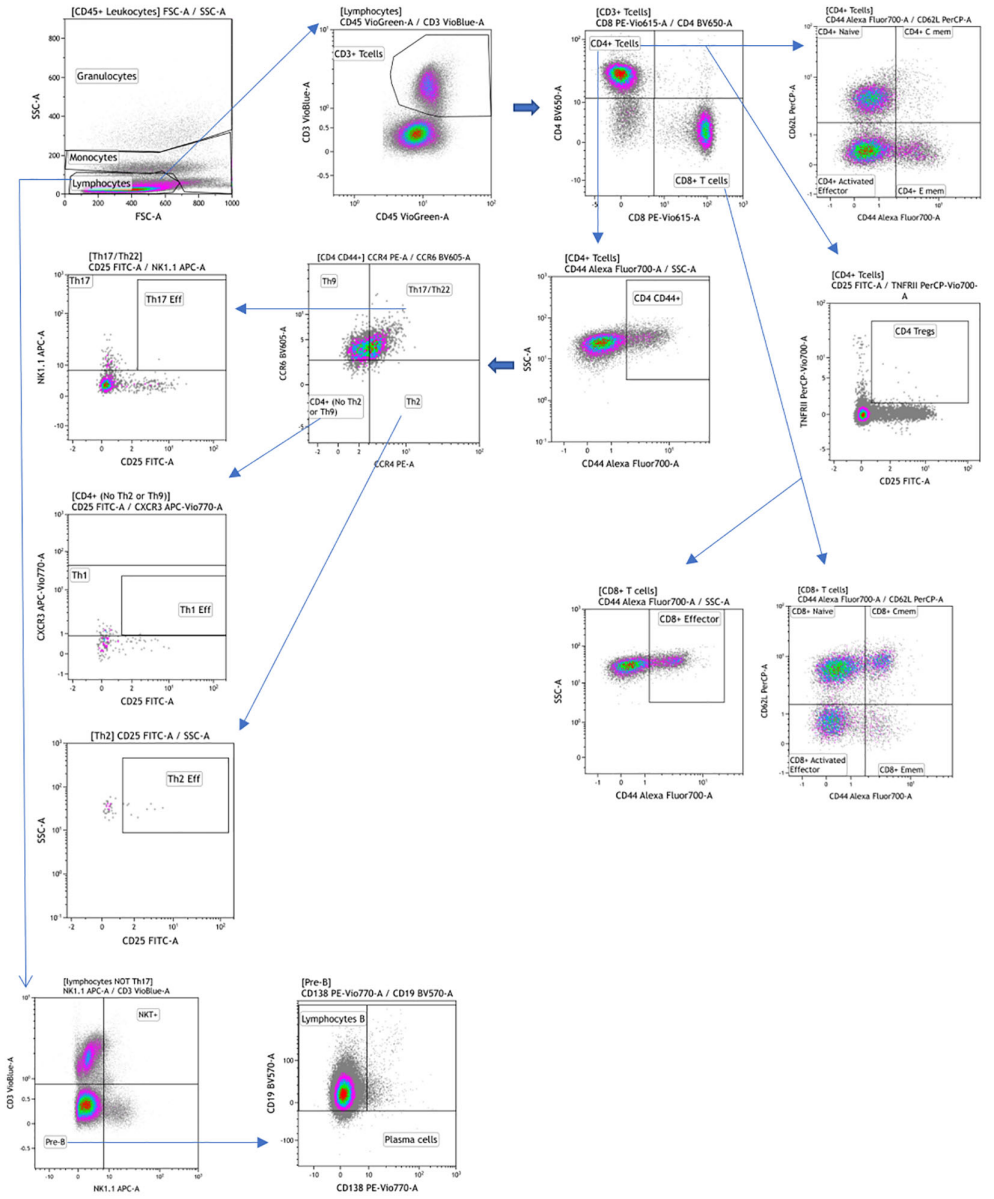
Manual gating strategy by differential expression of extracellular markers for the lymphoid panel. Representative examples of flow cytometry plots determined on whole blood labeled from one individual.

For the Myeloid and Lymphoid panels, fresh peripheral blood was labeled with a mixture of surface antibodies (**Table 1**) and incubated at room temperature for 30 minutes. After surface labeling, red blood cells (RBC) were lysed using the RBC Lysis/Fixation Buffer (BioLegend, San Diego, CA, USA). Antibody labeling for these panels was performed according to the reagents listed in **Table 1**, providing visualization of the main leukocyte populations (**Figures 1**, **2**).

For the Intracellular Panel, leukocytes were isolated after RBC lysis using the RBC Lysis Buffer (Miltenyi Biotec, Bergisch Gladbach, Germany). The lysate was removed, and the cells were washed with a staining buffer containing PBS and 2% fetal bovine serum without complement. Cell counts were determined using a Neubauer counting chamber. Approximately 0.5-1*10^6^ cells were labeled with surface antibodies (see **Table 2**). For intracellular labeling, cells were fixed and permeabilized using the FOXP3/Transcription Factor Staining Buffer Set (eBioscience, San Diego, CA, USA). To saturate excess protein-binding sites on cell membranes, cells were blocked with bovine serum albumin (BSA; Sigma Aldrich, St. Louis, Missouri, USA). After blocking, the cells were incubated with intracellular antibodies (see **Table 2** for details). The gating strategy used for this panel is described in **Supplementary Figure 1**.

### 2.4 Validation experiments design

To validate our proposed cytometry panels, we conducted one experiment using a model of acute lung inflammation (n=12). We also conducted another one to assess baseline variability (n=5) of immune subpopulations. A third assessment examined the impact of pre-experimental animal acclimatization to handling on intersubject variability. The specific protocols for each experiment are detailed below.

Animals were housed under constant ambient temperature conditions with a natural light cycle (12-hour photoperiod). They were provided ad libitum access to standard diet and water. Upon arrival at our facilities, the animals underwent a one-week acclimatization period to ensure they were well-adjusted to the new environment before the start of the experiments.

1) Validation of Peripheral Blood panels in an acute lung inflammation model: In this experiment, 12 female mice with a mean weight of 18.25 ± 1.04 g (Charles River) were used and randomly divided into 3 blocks of 4 animals, each one containing 2 lipopolysaccharide (LPS) animals and 2 control animals, thus totalizing 6 animals per group. The LPS group was administered 5 mg/kg of LPS (Sigma-Aldrich, Ref: L2880) in 100 µL of saline through intra-tracheal instillation (18), while control group received 100 µL of saline via intra-tracheal instillation. Blood samples from each animal were collected at three time points: at baseline (prior to LPS administration), at 24 hours (Myeloid and Lymphoid panels, were applied; **Table 1**), and at 72 hours post-induction upon sacrifice (Myeloid, Lymphoid and Intracellular panels were applied (**Tables 1**, **2**).

Clinical signs such as dehydration, piloerection, cleanliness, lesions, aggressiveness, passivity, and repetitive behaviors were monitored daily. Each of these variables was scored on a scale from 0 (no severity) to 4 (high severity). An endpoint criterion was set such that if the cumulative score exceeded 16 points, or if any single variable scored 4, the animal would be sacrificed.

2) Intrinsic Variability Assessment: Five male mice with a mean weight of 27.6 ± 2.59 g were used in this study. Blood samples were collected from each animal at five different time points: on days 1, 5, 8, 12, and 15. This repeated sampling was designed to evaluate both interindividual and intraindividual variability of immune subpopulations in peripheral blood.

3) Animal Handling Acclimatization: To determine the possible impact of animal acclimatization to handling on variability, baseline peripheral blood immune values were compared between the following two groups: the pre-LPS baseline values from the first experiment (after 10 days of pre-experimental handling) and day 1 values from the second experiment (without any prior handling). This comparison aimed to identify potential differences attributable to handling and related stress reduction. Pre-experimental handling was carried out for 10 days before starting and consisted on placing the animals into the anesthetic cage (without anaesthetizing them), introducing the operator’s hands into the cage to familiarize the animals with the smell, and finally returning them to their cages.

### 2.5 Data analysis

Flow cytometry data were analyzed using classical manual gating to identify known subpopulations, as detailed in **Figures 1**, **2**; **Supplementary Figure 1**. The analysis was performed using Kaluza software (version 2.1, Beckman Coulter, Brea, CA, USA). To ensure unbiased results, all data files were encrypted and analyses were conducted in a blinded fashion.

To quantify immune subsets, cell counts were normalized to cells per microliter (cells/µL) using the following equation:


= (Gate count cells/acquired volume) * (sample volume)

These absolute measurements allow for precise comparisons between different samples and experiments, accommodating variations in blood volume and providing a standardized metric for analyzing immune cell populations. For graphical representation, the absolute number was transformed to logarithm in base 10.

Statistical Analysis

Statistical analyses were performed using BlueSky Statistics software [v10.3.1, BlueSky Statistics LLC, Chicago, IL, USA]. The threshold for statistical significance was set at p ≤ 0.05 for all analyses. Our data did not violate assumptions of normality and homogeneity of variances.

To analyze variability data, we used repeated-measures analysis of variance (ANOVA) to evaluate individual changes over time and across different animals. The effect size (η²) was calculated to estimate the proportion of variance attributable to each factor, intra and interindividual. Additionally, the minimum detectable effect size was calculated assuming a design with an 80% statistical power and a Cohen’s d of 0.5 for each variable (medium size effect).

In the LPS validation study, the percentage of variation between the 24-hour sample and the baseline sample was calculated. Subsequently, Student’s t-tests were used to assess differences between the control and LPS-treated groups. Regarding the block design, we firstly carried out a two-way ANOVA with block and group as factors, showing no statistical significance of the block factor, so it was ignored in subsequent analysis. To determine whether pre-experimental animal acclimatization to handling influenced intersubject variability, an F-test for comparing variances was conducted.

## 3 Results

### 3.1 Validation of peripheral blood panels in an acute lung inflammation model

None of the mice in the experiment reached the predefined clinical endpoint criteria, although one mouse died before the endpoint was reached.

**Figure 3:**
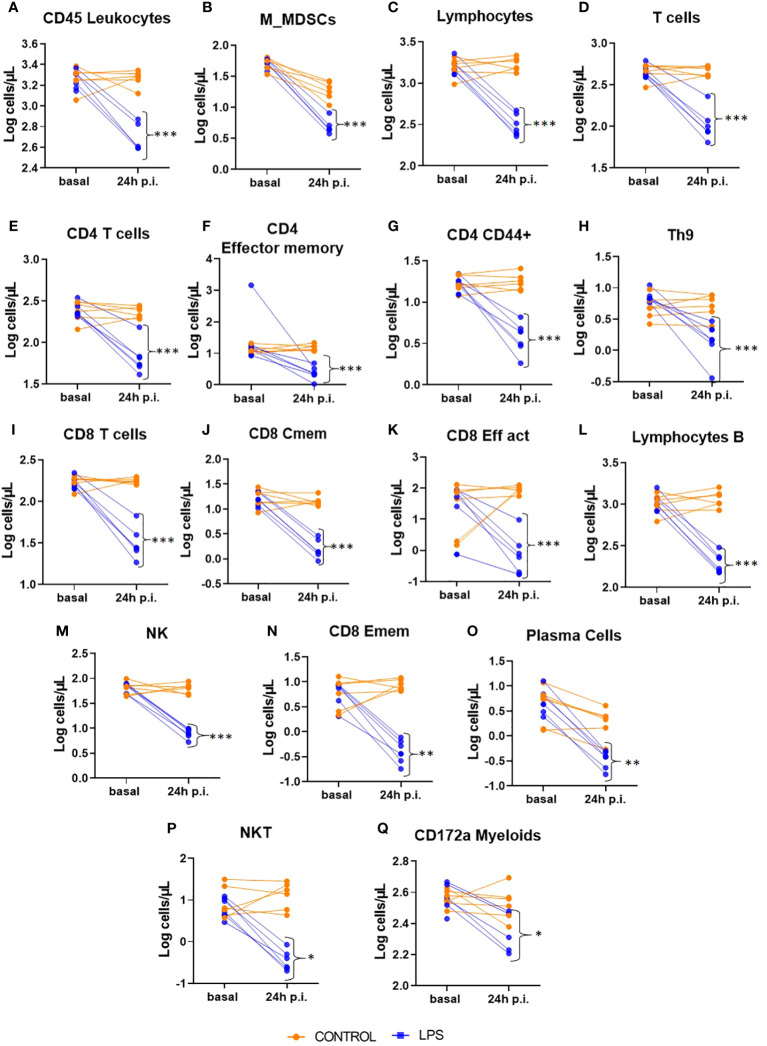
Intracellular panel at 72 hours post-injection (p.i.). This figure shows relative counts from the Intracellular panel in animals treated with LPS and in control animals at 72 hours post-injection. Only populations with significant differences between groups are presented. Significance levels are indicated as follows: *p ≤ 0.05; **p ≤ 0.01; ***p ≤ 0.001. **(A)** CD45 Leukocytes; **(B)** Monocyte type myeloid derived suppressor cell; **(C)** Lymphocytes; **(D)** Tcells; **(E)** CD4 Tcells; **(F)** CD4 Effector memory; **(G)** CD4 CD44 positive; **(H)** T Helper 9 Cells; **(I)** CD8 Tcells; **(J)** CD8 Central memory; **(K)** CD8 Effector activated; **(L)** lymphocytes B; **(M)** Natural Killers; **(N)** CD8 Effector memory; **(O)** plasma cells; **(P)** Natural killer T cell; **(Q)** CD172a myeloids cells.

Blocking strategy had no significant effect in none of the measured variables (data not shown). At 24 hours post-injection of LPS, there was no significant difference in the absolute numbers of neutrophils between the LPS-treated group and the control group. However, there was a significant reduction in all leukocytes and myeloid cells in the LPS group compared to controls (**Figures 3A, Q**). Notably, monocytic myeloid-derived suppressor cells (M-MDSCs) showed a pronounced decrease in the LPS group relative to the control group (**Figure 3B**).

Similarly, the lymphoid lineage populations exhibited significant reductions in the LPS group compared to the control group (**Figure 3C**). This reduction was observed across various immune cell types, including all T cells (**Figure 3D**), CD4+ T cells (and its effector and effector memory subsets) (**Figures 3E-G**), Th9 cells (**Figure 3H**), CD8+ T cells (along with its central memory and effector memory subsets) (**Figures 3I-K, N**), B lymphocytes (**Figure 3L**), plasma cells (**Figure 3O**), natural killer (NK) cells (**Figure 3M**), and natural killer T (NKT) cells (**Figure 3P**).

**Figure 4:**
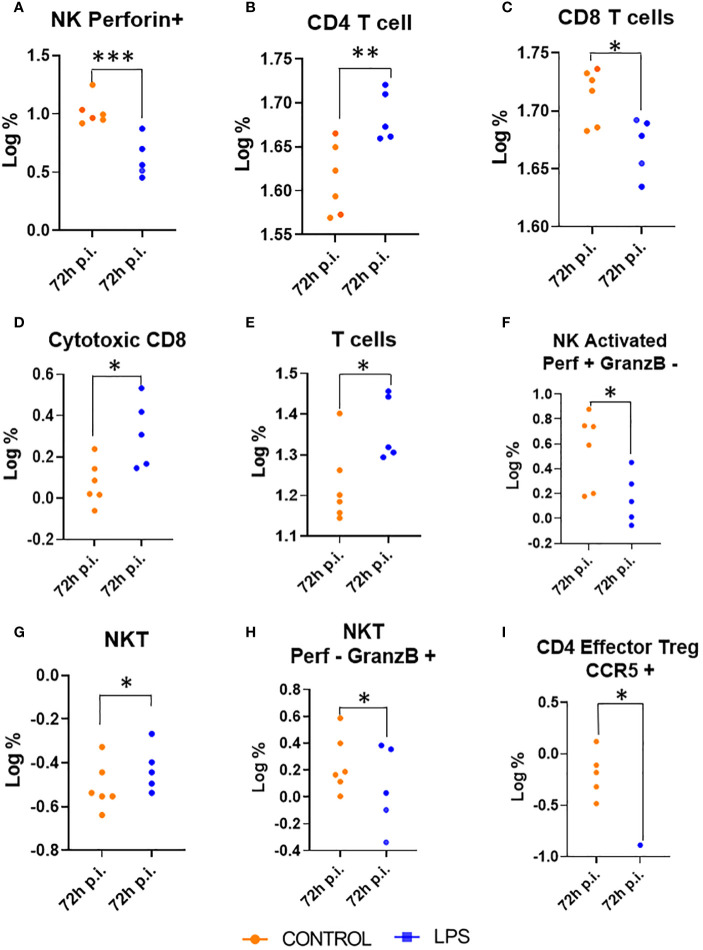
Changes in myeloid and lymphoid populations at 24 Hours Post-Injection (p.i.). This figure illustrates the absolute numbers of myeloid and lymphoid populations in animals treated with LPS and in control animals, measured 24 hours post-injection. Only the populations with significant differences between the two groups are displayed. Significance levels are indicated as follows: *p ≤ 0.05; **p ≤ 0.01; ***p ≤ 0.001. **(A)** Natural killer perforin; **(B)** CD4 Tcell; **(C)** CD8 Tcells; **(D)** Cytotoxic CD8; **(E)** Tcells; **(F)** Natural killer activated perofin positive and granzyme B negative; **(G)** Natural killer T cell; **(H)** Natural killer T cell perofin negative and granzyme B positive; **(I)** CD4 Effector Regulatory T cell CCR5 positive.

At 72 hours post-injection, the intracellular panel revealed notable changes in immune cell populations. In the LPS group, there was an increase in the percentage of all T cells (**Figure 4E**), CD4+ T cells and NKT cells compared to the control group (**Figures 4B, G**). Conversely, the CD8+ T cell population showed a decrease in percentage in the LPS group relative to controls (**Figure 4C**). Despite this reduction, the subset of CD8+ T cells with a cytotoxic phenotype, characterized by Granzyme B and perforin expression, increased in the LPS group compared to controls (**Figure 4D**).

In contrast, the LPS group showed a decrease in NK cells expressing perforin and its activated state (**Figures 4A, F**), NKT cells expressing granzyme (**Figure 4H**), and effector regulatory CD4+ T cells expressing CCR5 compared to the control group (**Figure 4I**).

In summary, 24 hours after administration intratracheal instillation of LPS we observed a significant decrease in nearly all immune cell populations compared to control group, with the exception of neutrophils, which remained unaffected. However, by 72 hours post-injection, there was a marked increase in populations with cytotoxic functions. Specifically, the subset of CD8+ T cells expressing cytotoxic markers Granzyme B and perforin increased, indicating a shift towards cytotoxic immune responses. Together with this increase in cytotoxic activity, there was a corresponding decrease in other immune cell subsets, including NKT cells expressing granzyme, NK cells expressing perforin, and effector regulatory CD4+ T cells expressing CCR5.

### 3.2 Intrinsic variability assessment

In this experiment, we analyzed intersubject and intrasubject variability of peripheral blood immune populations in healthy animals. Variance components associated with repetitions (intraindividual) and inter-individual variation were assessed for each variable by calculating the corresponding η² values (the proportion of variance explained by each factor). Interestingly, average intrasubject variability was twice that of intersubject variability in both panels (**Table 3**).

**Table 3:** Proportions of variance (ƞ2) associated with intra- and inter-individual factors.

Myeloid Panel			Lymphoid Panel		
Variables	η² Intra factor (%)	η² Inter factor (%)	Variables	η² Intra factor (%)	η² Inter factor (%)
**CD45_Leukocyites**	35.15	23.24	**Limphocytes**	37.41	34.31
**CD45_Myeloid**	54.53	12.70	**CD3_Tcells**	31.01	37.26
**CD172a_Myeloids**	42.25	24.21	**CD4_Tcells**	34.89	34.37
**Granulocytes**	27.37	32.33	**CD4_Naive**	49.38	5.53
**Basophils**	33.86	19.03	**CD4_act_eff**	48.64	14.63
**Neutrophils**	41.48	23.15	**CD4_Cmem**	36.90	18.04
**Eosinophils**	18.17	48.83	**CD4_Emem**	60.87	18.62
**Classical** **_Monocytes**	56.67	17.54	**CD4_CD44**	53.81	13.46
**Non_classical** **_monocytes**	41.12	34.31	**Th1**	57.32	11.61
**Total Monocytes**	28.68	45.25	**Th1_Eff**	32.73	29.35
**p_DC**	64.68	4.88	**Th2**	65.73	11.52
**Mature_pDC**	51.10	2.94	**Th2_Eff**	21.74	32.34
**cDC1**	16.86	33.11	**Th9**	65.74	12.99
**mature_cDC1**	16.88	16.88	**Th17**	77.49	3.97
**PMN_MDSCs**	19.04	13.77	**Th17_Eff**	49.79	14.30
**M_MDSCs**	20.03	14.22	**CD4+ Treg**	52.32	17.97
			**CD8_T_cells**	23.99	48.13
			**CD8_Naive**	45.53	10.11
			**CD8_act_eff**	43.55	17.96
			**CD8_Cmem**	16.57	54.62
			**CD8_Emem**	15.42	32.61
			**CD8_CD44+**	29.49	33.52
			**B Limphocytes**	20.16	45.08
			**Plasma_Cells**	9.80	76.16
			**NKT**	41.20	14.93
			**NK**	31.41	37.10

To facilitate the calculation of sample size for experiments involving these immunological variables, we calculated the minimum change necessary (both absolute and relative values) in the number of cells required to detect a medium effect size (Cohen’s d = 0.5) as significant (**Table 4**).

**Table 4:** Effect size corresponding to a Cohen’s d of 0.5 for the overall variables studied in the myeloid and lymphoid panels.

Variables	Detectable change threshold	Variables	Detectable change threshold
**CD45_Leukocytes**	363.44 (11.68%)	**Lymphocytes**	347.58 (16.28%)
**CD45_Mieloid**	148.52 (17.41%)	**CD3_Tcells**	93.22 (18.66%)
**CD172a_Myeloids**	103.11 (14.71%)	**CD4_Tcells**	40.92 (17.72%)
**Granulocytes**	80.04 (14.07%)	**CD4_Naive**	37.00 (49.58%)
**Basophils**	6.043 (19.04%)	**CD4_act_eff**	34.51 (27.13%)
**Neutrophils**	39.93 (18.51%)	**CD4_Cmem**	1.44 (33.93%)
**Eosinophils**	19.40 (19.47%)	**CD4_Emem**	4.93 (19.85%)
**Classical_Monocytes**	1.06 (32.65%)	**CD4_CD44**	3.90 (15.93%)
**Non_classical_monocytes**	2.89 (27.97%)	**Th1**	0.25 (32.26%)
**MONOCYTES**	36.48 (21.90%)	**Th1_Eff**	0.03 (37.22%)
**p_DC**	0.28 (38.09%)	**Th2**	0.23 (54.015%)
**Mature_pdc**	0.17 (38.34%)	**Th2_Eff**	0.05 (44.73%)
**cDC1**	0.25 (29.39%)	**Th9**	2.44 (27.66%)
**mature_CDc1**	0.07 (30.84%)	**Th17**	0.33 (46.06%)
**PMN_MDSCs**	3.59 (19.48%)	**Th17_Eff**	0.07(57.08%)
**M_MDSCs**	14.68 (16.23%)	**CD4+ Treg**	0.18(32.77%)
		**CD8_T_cells**	38.82(20.14%)
		**CD8_Naive**	31.85(37.40%)
		**CD8_act_eff**	28.65(37.44%)
		**CD8_Cmem**	5.50(25.24%)
		**CD8_act_eff**	28.65 (37.44%)
		**CD8_Cmem**	5.50 (25.24%)
		**CD8_Emem**	2.85 (30.89%)
		**CD8_CD44**	0.50 (29.56%)
		**Lymphocyes_B**	291.87 (20.50%)
		**Plasma_Cells**	10.11 (56.15%)
		**NKT**	14.13(26.59%)
		**NK**	13.38 (19.05%)

Thresholds for each population are represented as the number of cells and their relative percentage to total leukocytes (CD45+ cells).

### 3.3 Animal handling acclimatization

In the experiment to assess the impact of animal handling on intersubject variability, our results showed a reduction in variability in 8 out of 40 immune subsets studied, while 6 subsets showed an increase in their variability (**Supplementary Table 3**). Overall, we cannot state that there is a beneficial effect of handling on data variability.

## 4 Discussion

The cytometry panels developed in this study allow for extensive characterization of a wide range of immune subpopulations in mice, utilizing minimal peripheral blood volumes. This is especially useful for conducting longitudinal studies without adversely impacting the animal’s physiology. Besides, this approach aligns well with the 3R principles, reducing and refining animal experimentation, thanks to the use of very small blood samples and a 16-channel cytometer. To our knowledge, no similar methodologies for longitudinally analyzing immune subpopulations on such small blood volumes have been documented in the literature.

For instance, most research studies involving peripheral blood cytometry were conducted at the endpoint, with the animal’s entire circulating blood volume collected for various flow cytometry panels or other techniques such as immunohistochemistry (19–21). Despite the complexity of the immune response, involving multiple interrelated lineages, often results in flow cytometry studies only focus on major immune populations, such as total lymphocytes (22), or specific immunological parameters like cytokines (23). Even studies focusing on isolated organs (such as lung (24), spleen (25), liver (26), etc.) often examine a limited number of immune subpopulations.

The novelty of our method resides on the use of a highly optimized combination of 14 murine immune markers per panel. Thus, we are able to study 42 immune subsets thanks to a careful selection and combination of the 14 antibodies per panel. Fluorochromes selection process was carried out considering the antigen density and the fluorochrome spectral overlap. Previous studies that used fewer markers (20, 27) or equipment with limited fluorescence channels (21) could analyze fewer populations. The limited commercial availability of conjugated antibodies to different fluorophores in mice (8) further complicates the design of complex panels. Additionally, many preclinical immunological studies report results only as percentages, due to the need for washing, that limits their ability to obtain absolute cell counts. Our approach, which uses very small blood volumes without needing counting microbeads, allows obtaining absolute values for numerous subpopulations, thus better aligning with the standard practice in clinical studies (28, 29).

The immune panels defined in this study were validated using an acute model of lipopolysaccharide (LPS)-mediated lung inflammation. This model was chosen for its biological relevance, cost-effectiveness, and reproducibility (22, 30, 31). Therefore, our aim was to validate our panels, not focusing on fully describing the inflammatory processes observed. Our results show that these immunological panels are sensitive enough to detect changes in peripheral blood at 24 hours post-LPS induction. The observed decrease in circulating immune populations in the LPS group may be due to cell migration to inflamed lung tissue, consistent with findings from other studies in different mouse strains (22, 32). This suggests that our panels are sensitive and complete enough for flow cytometry-based studies of circulating immune populations in various experimental immune-related mice models.

Our study quantifying inter- and intra-subject components provides insights into the sources of natural variability in preclinical studies involving mice. This may help to better define peripheral blood flow cytometry experimental designs, considering the relative importance of inter-individual and intraindividual (repetition) factors. Although few studies have evaluated the variability of the immune system over time in healthy animals (33), our findings align with some reports showing that myeloid populations exhibit greater variability than lymphoid populations (34), likely due to their lower abundance.

Interindividual variability is a significant source of variation, impacting the statistical power and reproducibility of animal experiments (35). Nevertheless, our study reveals that intraindividual variance is more relevant than interindividual one. This finding suggests that individual differences within the same subject over time can be substantial, and researchers should meticulously account for them during experimental design and data analysis. Although the inter-subject variability effect has been considered in other studies (35, 36), our current understanding suggests that it has primarily been explored within the context of neuroscience and behavioral-related experiments. Our results also show that inter- and intrasubject variability seem to affect immune populations differently. Thus, in order to help in the design of future experiments, we have quantified the minimum change necessary to detect a medium effect size on each immune subpopulation (**Table 4**). Based on our current understanding, no previous reports have provided this type of statistical information.

Finally, we have tried to evaluate the influence on variability of pre-acclimatization of animals to handling. In disagreement with previous works (37, 38), we have failed to find a relevant effect on variability reduction of pre-experimental acclimatization to animal handling. This could be due to the measurement of behavioral rather than physiological variables in these previous studies. Nevertheless, our results agree with other references about handling acclimatization, in which researchers consider that repeated handling exposure may even worsen animal stress (39).

Our study suffers from several limitations. First, the small sample size in all the experiments might have prevented us from finding small effects or effects on highly variable subpopulations. Nevertheless, it aligns with the 3Rs principles of reducing sample sizes in animal experimentation, and our sample size is in agreement with some previous immunological studies with LPS (40, 41). Second, our study was conducted on a single mouse strain, so we cannot guarantee that our flow cytometry protocol might require slight adaptations for other strains. Third, bronchoalveolar lavage was not performed to confirm cell migration to the lung in LPS-treated animals, thus leaving room for further investigation about the causes of immune subpopulations shrinking after the LPS insult. No attempt to compare with previous methodologies has been made. This is due to the fact that most of previous approaches did not allow for longitudinal assessment (they require animals sacrifice) or measures a very limited set of parameters (21, 31, 32). Similarly, no attempt has been made to characterize other sterile or septic inflammatory models. Although this characterization with our comprehensive panels may be of interest, it is beyond the scope of this paper. Lastly, the use of non-spectral cytometers with less than 14 channels would prevent the use of the panels we have developed, thus limiting their applicability scope.

In conclusion, our novel panels allow a comprehensive study of the murine immune system with minimal blood volumes, facilitating the use of absolute and relative percentages in peripheral blood studies. For the first time, variance components in healthy animals have been characterized, providing a better foundation for estimating the number of animals needed to detect immune changes with high statistical power, and we have not detected a relevant effect of pre-experimental handling on variability. These results may provide a robust platform for longitudinal immunological studies in mice and can be used to uncover significant insights into immune responses.

## Data Availability

The raw data supporting the conclusions of this article will be made available by the authors, without undue reservation.
