# Supplementary material for: Design and validation of novel flow cytometry panels to analyze a comprehensive range of peripheral immune cells in mice
Source: Front Immunol. 2024 Aug 13;15:1432816. doi: 10.3389/fimmu.2024.1432816 (PMC11350558; doi:10.3389/fimmu.2024.1432816)
Supplement: Supplementary file 1 [file DataSheet1.pdf]

Supplementary material

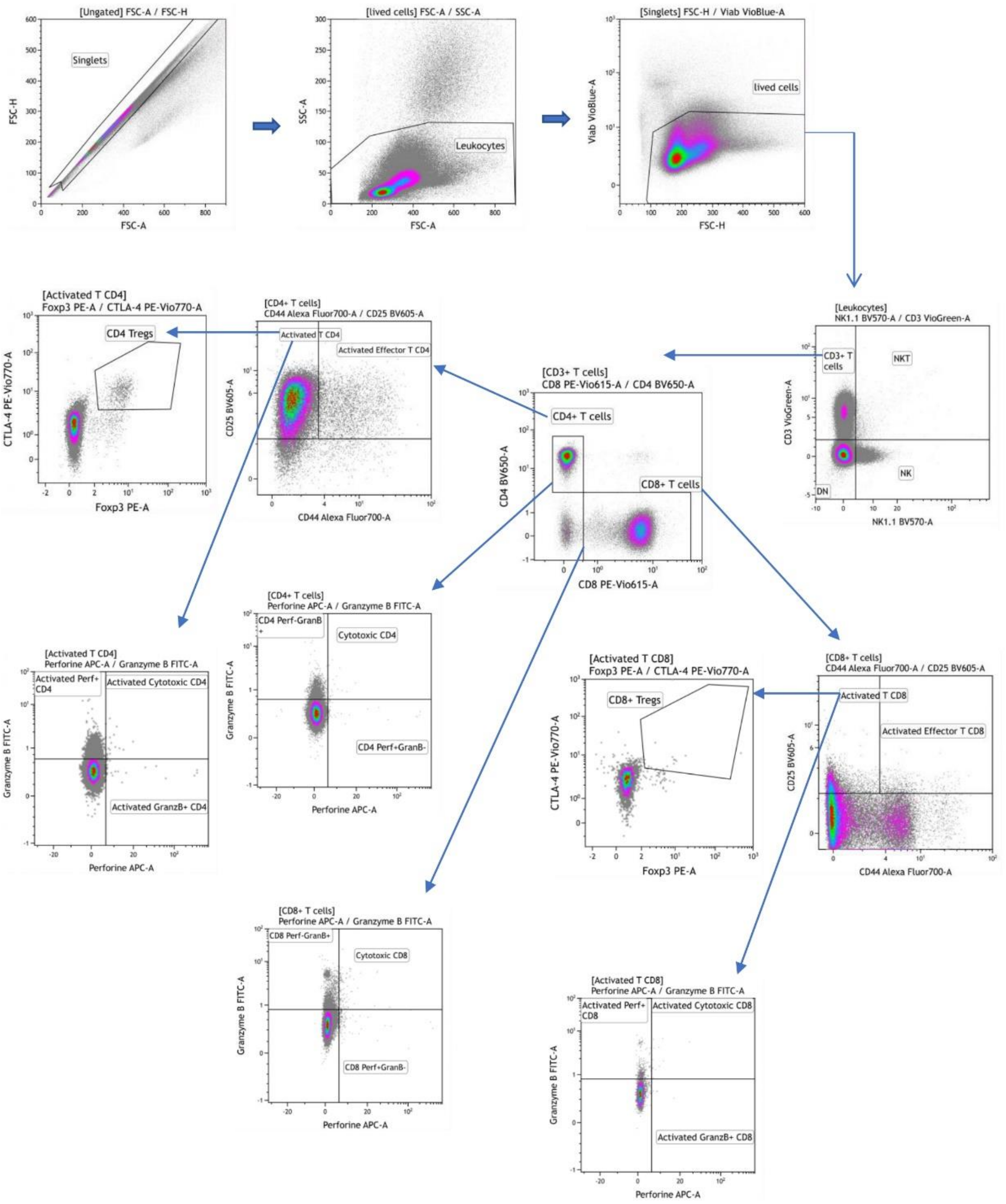

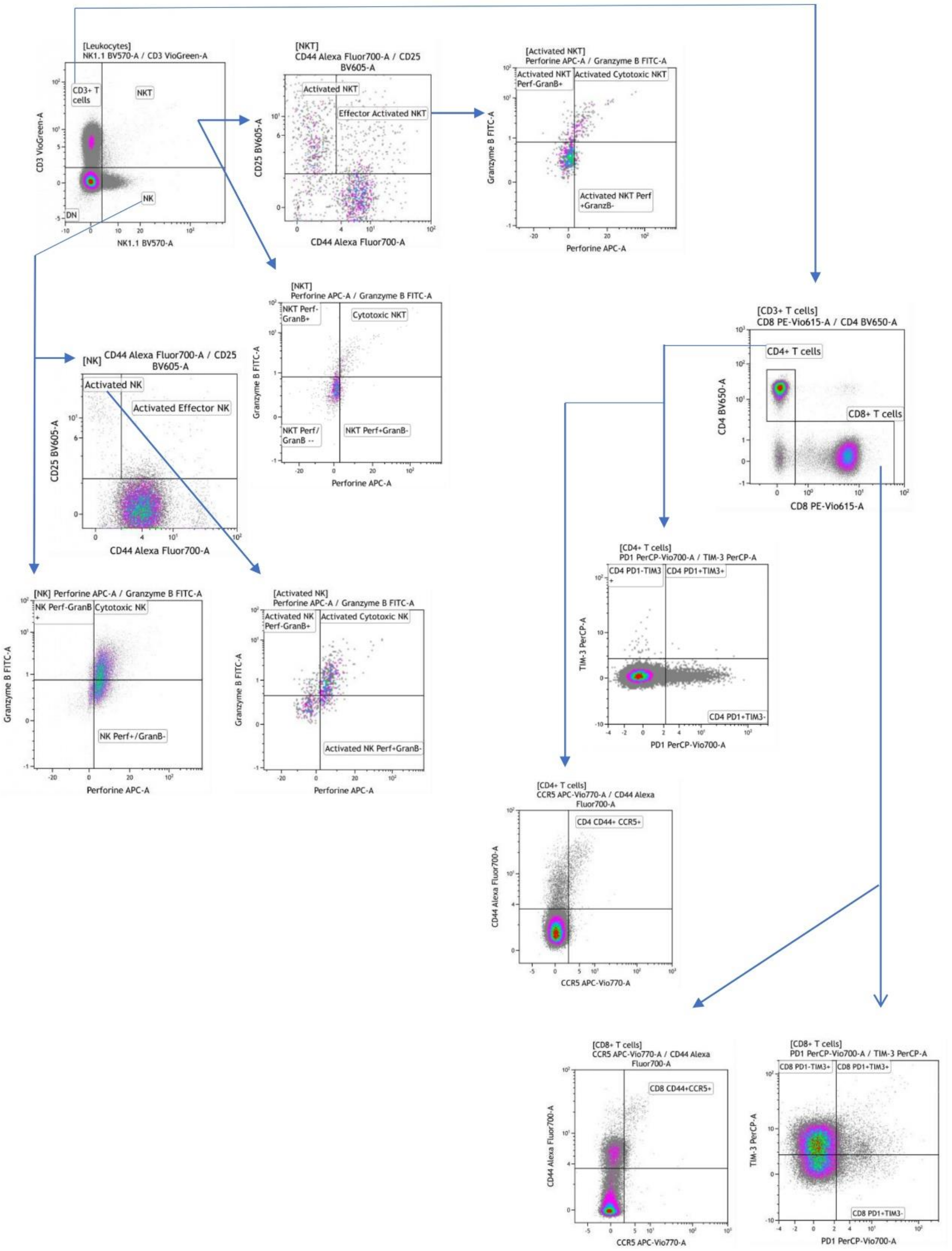
[Leukocytes]
NK1.1 BV570-A / CD3 VioGreen-A
CD3+ T cells
NKT
DN
NK
CD3 VioGreen-A
NK1.1 BV570-A
[NKT]
CD44 Alexa Fluor700-A / CD25 BV605-A
Activated NKT
Effector Activated NKT
CD25 BV605-A
CD44 Alexa Fluor700-A
[Activated NKT]
Perforine APC-A / Granzyme B FITC-A
Activated NKT Perf-GranB+
Activated Cytotoxic NKT
Activated NKT Perf +GranzB-
Granzyme B FITC-A
Perforine APC-A
[NKT]
Perforine APC-A / Granzyme B FITC-A
NKT Perf-GranB+
Cytotoxic NKT
NKT Perf/ GranB --
NKT Perf+GranB-
[NK]
CD44 Alexa Fluor700-A / CD25 BV605-A
Activated NK
Activated Effector NK
[NK] Perforine APC-A / Granzyme B FITC-A
NK Perf-GranB +
Cytotoxic NK
NK Perf+/GranB-
[Activated NK]
Perforine APC-A / Granzyme B FITC-A
Activated NK Perf-GranB+
Activated Cytotoxic NK
Activated NK Perf+GranB-
[CD3+ T cells]
CD8 PE-Vio615-A / CD4 BV650-A
CD4+ T cells
CD8+ T cells
CD4 BV650-A
CD8 PE-Vio615-A
[CD4+ T cells]
PD1 PerCP-Vio700-A / TIM-3 PerCP-A
CD4 PD1-TIM3 +
CD4 PD1+TIM3+
CD4 PD1+TIM3-
TIM-3 PerCP-A
PD1 PerCP-Vio700-A
[CD4+ T cells]
CCR5 APC-Vio770-A / CD44 Alexa Fluor700-A
CD4 CD44+ CCR5+
CD44 Alexa Fluor700-A
CCR5 APC-Vio770-A
[CD8+ T cells]
CCR5 APC-Vio770-A / CD44 Alexa Fluor700-A
CD8 CD44+CCR5+
[CD8+ T cells]
PD1 PerCP-Vio700-A / TIM-3 PerCP-A
CD8 PD1-TIM3+
CD8 PD1+TIM3+
CD8 PD1+TIM3-

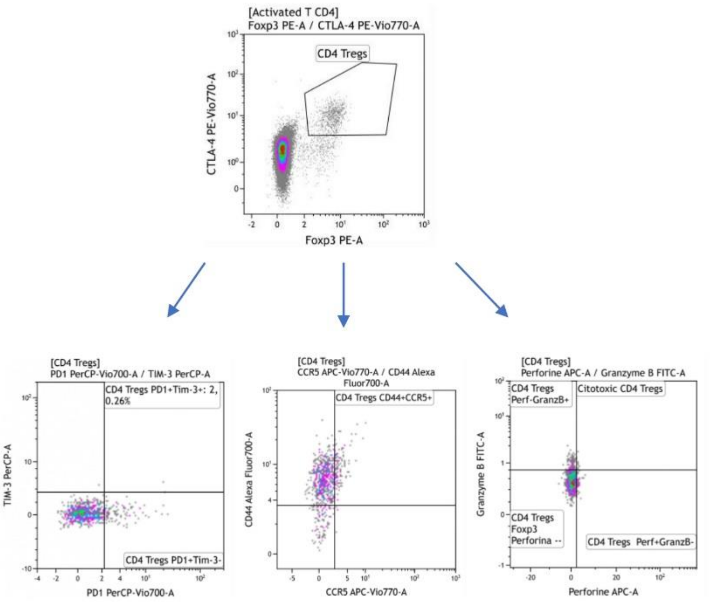

Figure S1. Manual gating strategy by differential expression of markers for the intracellular panel.

Representative examples of flow cytometry plots determined on whole blood labeled from one individual

Table S1.

Titration of antibodies used in the myeloid and lymphoid panels. Selected titration volume is marked in bold type

**Myeloid Panel**					
**Name**	**Channel**	**Comercial brand**	**Reference**	**Clone**	**Titration in 50 µL**
B220	APC5	Biolegend	103231	RA3-6B2	**0.5 µL**<br>0.7 µL
CCR2	PC7	Miltenyi	130-120-818	REA538	0.5 µL<br>0,7 µL<br>**1 µL**
CD11b	PC5.5	Miltenyi	130-113-809	REA592	**0.5 µL**<br>1 µL<br>1.5 µL
CD11c	APC7	Miltenyi	130-110-704	REA754	0.5 µL<br>0.7 µL<br>**1 µL**
CD172a	VioBlue	Miltenyi	130-123-151	REA1201	**0.5 µL**<br>1µL
CD45	VioGreen	Miltenyi	130-123-900	30F11	**1 µL**<br>2.5µL
CD49b	ECD/PE-Vio615	Miltenyi	130-116-437	REA981	0.5 µL<br>**1 µL**
Ly6C	PerCP/PC5	Biolegend	128028	HK1.4	0.5 µL<br>**0.7 µL**<br>1 µL
CD80	APC	Miltenyi	130-116-461	REA983	0.5 µL<br>0.7 µL<br>**1 µL**
CD86	APC	Miltenyi	130-102-558	PO3.3	1 µL<br>2 µL<br>**2.5 µL**
F4/80	FITC	Miltenyi	130-117-509	REA126	**0.5 µL**<br>0.75 µL<br>1 µL
Ly6G	BV605	Biolegend	127639	1A8	0.5 µL<br>**0.7 µL**<br>1 µL
MHC-II	BV650	Biolegend	107641	M5/114.15.2	0.5 µL<br>**0.7 µL**<br>1 µL
Siglec-F	PE	Miltenyi	130-112-174	REA798	0.5 µL<br>0.7 µL<br>**1 µL**
**Lymphoid Panel**					
**Name**	**Channel**	**Comercial brand**	**Reference**	**Clone**	**Titration in 50 µL**
CCR4	PE	Biolegend	131204	2G12	0.5 µL<br>1 µL<br>2 µL<br>**2.5 µL**
CCR6	BV605	Biolegend	129819	29-2L17	0.7 µL<br>1 µL<br>1.25 µL<br>**2.5 µL**
CD138	PC7	Miltenyi	130-102-318	REA104	**1 µL**<br>2.5 µL<br>5 µL

CD19	BV570	Biolegend	115535	6D5	0.7µL<br>**1 µL**<br>1.25 µL
CD25	FITC	Miltenyi	130-120-088	REA568	0.5 µL<br>0.7 µL<br>**1 µL**
CD3	VioBlue	Miltenyi	130-118-849.	17A2	0.5 µL<br>0.7 µL<br>**1 µL**
CD4	BV650	Biolegend	100545	RM4-5	0.7 µL<br>**1.25 µL**<br>2.5 µL
CD44	APC5	Biolegend	103026	IM7	**0.5 µL**<br>0.7 µL
CD45	VioGreen	Miltenyi	130-123-900.	30F11	0.5 µL<br>0.7µL<br>**1 µL**<br>2.5 µL
CD62L	PerCP/PC5	Biolegend	104410	MEL-14	0.5 µL<br>**0.7 µL**<br>1 µL
CD8	ECD/PE-Vio615	Miltenyi	130-123-914	REA601	0.5 µL<br>0.7 µL<br>**1 µL**
CXCR3	APC7	Biolegend	126540	CXCR3-173	0.5 µL<br>**0.7 µL**<br>1 µL
NK1.1	APC	Miltenyi	130-120-507	REA1162	0.5 µL<br>0.7 µL<br>**1 µL**
TNF-RII	PC5.5	Miltenyi	130-104-701	REA228	1 µL<br>**2 µL**<br>2.5 µL<br>5 µL

Table S2.

Titration of antibodies used in intracellular panel.Selected titration volume is marked in bold type.

**Name**	**Channel**	**Comercial brand**	**Reference**	**Clone**	**Titration in 10^6 cells**
Viability	VioBlue	ebiosciencie	65-0863-18		Non titrated
CD3	VioGreen /BV510	Biolegend	100233	17A2	**2.5 µL**<br>5 µL
NK1.1	BV570	Biolegend	108733	PK136	2.5 µL<br>**5 µL**
CD25	BV605	Biolegend	102035	PC61	2.5 µL<br>**5 µL**
CD4	BV650	Biolegend	100545	RM4-5	0.7 µL<br>1.25 µL<br>**2.5 µL**
Granzyme B	FITC	Biolegend	372205	QA16A02	Non titrated
FOXP3	PE	Miltenyi	130-111-678	REA788	Non titrated
CD8	ECD/PE-Vio615	Miltenyi	130-123-914	REA802	0.7 µL<br>**1 µL**<br>2 µL
TIM-3	PerCP/PC5	RD Systems	FAB1529C	215008	Non titrated
PD1	PC5.5	Miltenyi	130-111-957	REA802	0.7 µL<br>**1 µL**<br>2 µL
CTLA4	PC7	Biolegend	106313	UC10-4B9	Non titrated
Perforina	APC	Biolegend	154303	S16009A	Non titrated
CD44	APC5	Biolegend	103026	IM7	0.5 µL<br>0.7 µL<br>**1 µL**
CCR5	APC7	Miltenyi	130-120-168	REA354	0.7 µL<br>**1 µL**<br>2 µL

Table S3.

Handling effect evaluation on intersubject variability comparing baseline samples of LPS experiment (after 10 days of handling) and the variability experiment (without animal handling) using F-test Calculator.

**Variables**	P-value	Test statistic F
**Leukocytes**	0.66	0.71
**CD45_Myeloid**	0.003***	27.14
**CD172a_Myeloids**	0.02331*	10.6162
**Granulocytes**	0.024*	10.5
**Basophils**	0.5	1.9
**Neutrophils**	0.26	2.97
**Eosinophils**	0.34	2.5
**Classical _Monocytes**	0.6164	1.5911
**Non_classical _monocytes**	0.11	4.9
**Monocytes**	0.03*	10.1
**p_DC**	0.3273	2.5767
**cDC1**	0.0002*	76.1
**PMN_MDSCs**	0.9	1.1
**M_MDSCs**	0.04*	8.3

**Variables**	P-value	Test statistic F
**Lymphocytes**	0.57	0.54
**CD3_Tcells**	0.62	0.58
**CD4_Tcells**	0.17	0.22
**CD4_Naive**	0.0003***	0.007
**CD4_act_eff**	0.057	0.12
**CD4_Cmem**	0.007**	0.04
**CD4_Emem**	0.48	1.97
**CD4_CD44**	0.01**	5.3
**Th1**	0.02124*	0.06654
**Th1_Eff**	0.7	1.44
**Th2**	0.1676	3.9045
**Th2 Eff**	0.81	1.23
**Th9**	0.2815	2.847
**Th17**	0.0005***	0.01
**Th17_Eff**	0.008**	0.04
**Treg**	0.1101	0.1694
**CD8_T_cells**	0.06	1.63
**CD8_Naive**	0.005**	0.03
**CD8_act_eff**	0.16	0.21
**CD8_Cmem**	0.8458	0.7927
**CD8_Emem**	0.73	1.36
**CD8_EFF**	0.34	2.47
**B Limphocytes**	0.52	0.5
**Plasma_Cells**	0.01**	15.9516
**NKT**	0.88	1.13
**NK**	0.64	0.6
